# Structured illumination to spatially map chromatin motions

**DOI:** 10.1117/1.JBO.23.5.056007

**Published:** 2018-05-15

**Authors:** Keith Bonin, Amanda Smelser, Naike Salvador Moreno, George Holzwarth, Kevin Wang, Preston Levy, Pierre-Alexandre Vidi

**Affiliations:** a Wake Forest University, Department of Physics, Winston-Salem, North Carolina, United States; b Wake Forest School of Medicine, Department of Cancer Biology, Winston-Salem, North Carolina, United States; c Comprehensive Cancer Center of Wake Forest Baptist Medical Center, Winston-Salem, North Carolina, United States

**Keywords:** diffractive optics, laser photoactivation, particle tracking, diffusion maps, chromatin motion, structured illumination

## Abstract

We describe a simple optical method that creates structured illumination of a photoactivatable probe and apply this method to characterize chromatin motions in nuclei of live cells. A laser beam coupled to a diffractive optical element at the back focal plane of an excitation objective generates an array of near diffraction-limited beamlets with FWHM of 340±30  nm, which simultaneously photoactivate a 7×7 matrix pattern of GFP-labeled histones, with spots 1.70  μm apart. From the movements of the photoactivated spots, we map chromatin diffusion coefficients at multiple microdomains of the cell nucleus. The results show correlated motions of nearest chromatin microdomain neighbors, whereas chromatin movements are uncorrelated at the global scale of the nucleus. The method also reveals a DNA damage-dependent decrease in chromatin diffusion. The diffractive optical element instrumentation can be easily and cheaply implemented on commercial inverted fluorescence microscopes to analyze adherent cell culture models. A protocol to measure chromatin motions in nonadherent human hematopoietic stem and progenitor cells is also described. We anticipate that the method will contribute to the identification of the mechanisms regulating chromatin mobility, which influences most genomic processes and may underlie the biogenesis of genomic translocations associated with hematologic malignancies.

## 1 Introduction

Dynamic motions of chromatin are thought to critically influence genomic processes such as gene expression, DNA replication, DNA repair, and the biogenesis of genomic translocations. Chromatin motions follow stochastic constrained random walks,^1^ which complicates the interpretation of their biological significance. Most studies on chromatin mobility rely on tracking artificial DNA arrays integrated in the genome,^2^^–^^4^ or more recently, DNA repeats by CRISPR/dCas9 imaging.^5^^,^^6^ These tracking approaches have several limitations. First, artificial DNA arrays do not fully reproduce the complex chromatin organization,^7^ and chromatin binding of the bulky dCas9-GFP reporter may generate a drag that could affect chromatin dynamics. Second, only a few measurements per cell are typically possible, from which a global assessment of chromatin dynamics is difficult. As such, new optical techniques are needed to interrogate a large number of regions in the cell nucleus, over meaningful periods of time. Photoactivatable histone probes have been used as an alternative to study chromatin motions in a near-native chromatin context.^8^^–^^10^ With this approach, the optical method used for photoactivation is critical, and conventional scanning confocal microscopy commonly used for analysis does not allow to simultaneously illuminate and photoactivate multiple subcellular regions.

Structured illumination has been increasingly applied over the last decades to improve performance in wide-field microscopy.^11^^,^^12^ In addition to superresolution microscopy, applications for structured illumination include optical trapping, surface profiling, quantitative phase imaging in biological systems, and optical sectioning achieved by illumination with incoherent light.^11^^,^^13^^,^^14^ Here, we apply structured illumination for simultaneous photoactivation of chromatin reporters throughout the nucleus of live cells. Our structured light pattern is produced with a diffractive optical element (DOE) module, implemented as a simple modification to a commercial inverted microscope system. By enabling parallel measurements in native chromatin environments, the approach circumvents the limitations discussed above. It yields robust values of diffusion at multiple points within a single cell, thereby reducing the need for agglomerating population-based measurements, and provides spatial information on chromatin motions.

## 2 Materials and Methods

### 2.1 Instrument Design

**Fig. 1:**
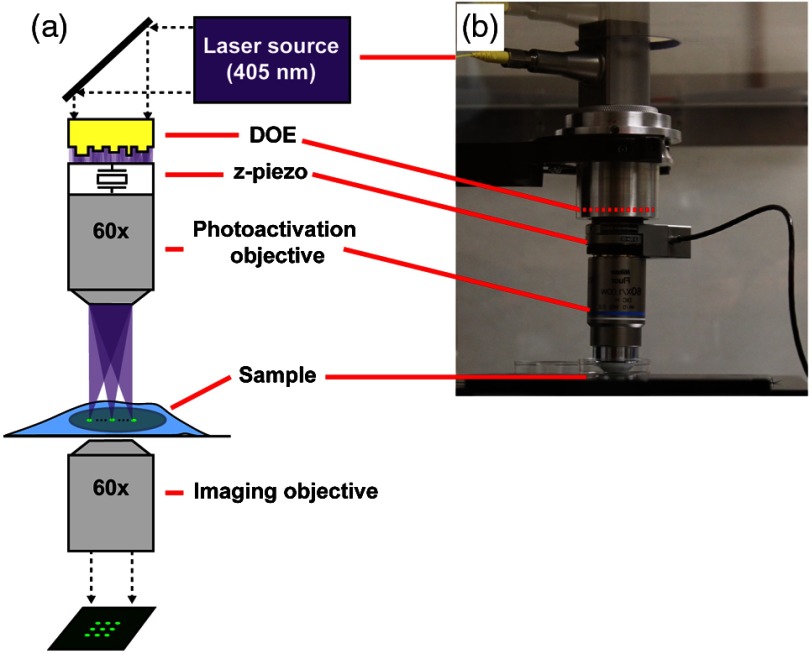
DOE photoactivation module. (a) Schematic of the instrumentation. (b) Photograph of the custom DOE module mounted on the condenser arm of an Olympus IX-83 inverted microscope.

The optical setup is shown in Fig. 1. The laser source is a 30-mW fiber pigtailed diode laser (Thorlabs LP405-SF30) producing light at a wavelength of 405 nm, the photoactivation wavelength of photoactivatible green fluorescent protein (PAGFP). The fiber is single mode to produce a clean Gaussian spatial profile that is collimated by a metal mirror (Thorlabs RC12FC-F01), oriented to produce a reflected beam at 90 deg to the incoming axis, and designed to produce a beam of 12 mm in diameter. The collimated beam reflected downward by the collimating mirror is transmitted through a fused quartz DOE (Holo/Or MS-571-S-Y-X) designed to produce a 7×7 pattern of near diffraction-limited spots with high efficiency (∼76%) at 405 nm. The DOE has a clear aperture of 22.9 mm and a thickness of 3 mm. The separation angle between the central ray of adjacent beamlets is 0.028×0.028  deg. This generates a pattern with a full angle of 0.17×0.17  deg. Both sides of the DOE are AR-coated for 405 nm, and the zero-order spot in the middle is specified to be 90% to 130% in intensity relative to the other spots, to ensure high uniformity. The width and timing of square pulses input to the modulation port on the laser power supply are controlled by an Agilent 3320A Waveform Generator. The upper objective used for photoactivation is a 60× Nikon water-immersion lens with NA=1.00 and a working distance of 2 mm. A z-axis piezo holding this objective is used to finely adjust the image plane location of the beamlets produced by the DOE. The DOE photoactivation module is mounted on the condenser arm of an IX83 inverted microscope (Olympus) with a custom adaptor. The lower imaging objective (60× Olympus oil-immersion, NA=1.35) is used to acquire epifluorescence images of photoactivated GFP. The fluorescence light source is an Olympus U-HGLGPS, which uses a 130-W mercury vapor short arc bulb and a fiber optic light guide to couple the source to the microscope. A GFP filter cube (470/40 EX; 525/5 EM; 495LP BS; set 49002, Chroma) separates excitation and emitted light. Images are recorded with a scientific CMOS camera (ORCA-FLASH 4.0 LT, Hamamatsu) with a 6.5  μm×6.5  μm pixel size. At 60× magnification, the conversion factor between pixels and distance at the sample is 9.27  pixels/μm.

### 2.2 System Characterization

**Fig. 2:**
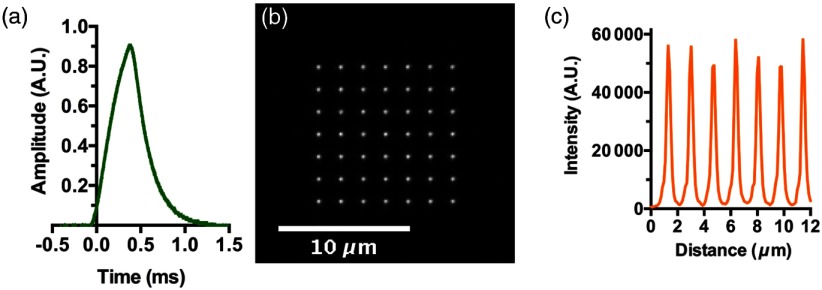
System characterization. (a) Time profile of the laser pulse produced after driving the 405-nm laser modulation port with a 0.5-ms square pulse. (b) Matrix of spots generated by the DOE module and directly visualized by bright field imaging, using only a #1.5 coverslip and water/oil-immersion media in the light path. (c) Intensity profile along one row of the spot matrix.

We used a Thorlabs microscope slide power sensor (S170C) and energy meter console (PM100D) to measure the power of the laser photoactivation pulse, and a photodiode (Thorlabs, DET200) to measure its temporal profile. The analysis showed that when the power supply of the 405-nm laser diode was controlled with a 0.5-ms square pulse (of 250 mV amplitude), the time-course of the emitted light exhibited a shark’s tooth pattern [Fig. 2(a)]. The peak power for the whole pattern was approximately 12 mW at the highest setting of driving amplitude. To estimate the photon flux in one of the 49 DOE-generated spots, we multiplied the peak power of 7.6 mW measured at the sample for the whole array of beams by the efficiency (0.76), and divided by the number of spots (49) to obtain ∼120  μW in a spot, corresponding to a photon rate of $R=1.8\times 10^{10}\text{ photons}/s$. Given a spot area of $∼0.78\;{\mu m}^{2}$, the photon flux $F=R/A=2.3\times 10^{10}\text{ photons}/{\mu m}^{2}\;s$ ($I=170\;\mathrm{mW}/{\mathrm{mm}}^{2}$). This number is well below the photon flux of $1\times 10^{12}\text{ photons}/{\mu m}^{2}\;s$ determined as a phototoxic threshold in eukaryotic cells for light of wavelength 488 nm.^15^

As an initial characterization of the spot array generated by the DOE module, images of the array were directly recorded using the brightfield setting of the microscope [Fig. 2(b)]. This setup was ideal in that no optical medium other than distilled water was in the light path. Under these conditions, individual spots were 340±30  nm in diameter, defined as the full-width at half-maximum of intensity [FWHM; Fig. 2(c)]. Using a ${\mathrm{TEM}}_{00}$ Gaussian laser beam radius, $\omega_{0}$, derived from the measured FWHM, we can estimate a theoretical depth of field using the corresponding confocal beam parameter, defined as $2\pi \omega_{0}^{2}/\lambda$.^16^ Substituting $\omega_{0}=\mathrm{FWHM}/\sqrt{2\;\ln\;2}=289\;\mathrm{nm}$ into the confocal beam parameter expression gives a theoretical depth of focus of 1.3  μm.

### 2.3 Image Preprocessing

**Fig. 3:**
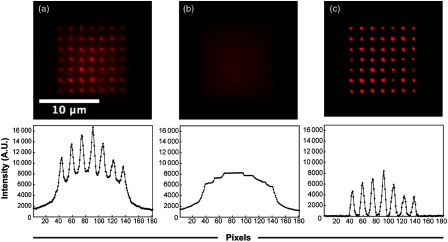
Image preprocessing. (a) Fluorescence image generated after photoactivation of the NVOC-RhQ dye in clear epoxy resin. (b) Background image (see text). (c) Background-subtracted NVOC-RhQ photoactivation image. Intensity profiles are shown below each image. Each dot corresponds to one pixel (equivalent to 0.1  μm).

We used a photoactivatable fluorescent dye (rhodamine Q caged with ortho-nitroveratryloxycarbonyl; NVOC-RhQ^17^) embedded in clear epoxy resin to test the DOE’s performance and to establish the image preprocessing method. After photoactivation with the 405-nm laser, an array of NVOC-RhQ spots was detected using a TxRed/rhodamine filter set [Fig. 3(a)].

The intensity profile across the photoactivated NVOC-RhQ dot matrix revealed a “pedestal” of fluorescence between spots compared to direct imaging in water, which may interfere with determination of spot position, in particular for images with low signal-to-noise. This background is likely caused by the scattering of 405-nm light in the nonideal epoxy medium, leading to NVOC-RhQ photoactivation under and between the intended spots. The pedestal was estimated by morphologically opening the raw image with a “rolling ball” structuring element^18^ [Fig. 3(b)]. The radius R and height H of the ellipsoidal structuring element were chosen to maximize the height of the peaks (our signal) and minimize the background in the background-subtracted image [Fig. 3(c)]. Values of 7 to 10 pixels for R worked well; not surprisingly, these values are about half of the distance between peaks (15.5 pixels). Since all spots have the same diameter, a single value of R (R=7) was used for all 49 spots. Satisfactory values of H were in the range of 2 to 10. H=10 was used. The pedestal was subtracted from the original image, yielding the background-subtracted image shown in Fig. 3(c). The FWHM of photoactivated NVOC-RhQ spots was 640±60  nm (n=5 frames). After background subtraction, each spot was fitted to a two-dimensional (2-D) Gaussian. Spot centers {x,y} derived from the fitting process were used to track spot motions.

## 3 Results

### 3.1 Chromatin Marker Photoactivation Within Fixed and Live Cells

**Fig. 4:**
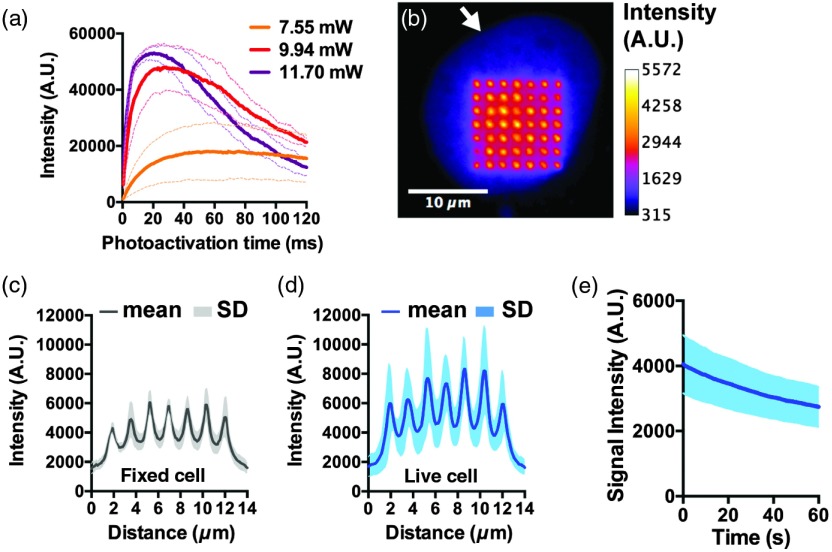
Chromatin marker photoactivation with DOE. (a) GFP emission curves in fixed U2OS cell nuclei after photoactivation with three levels of 405-nm laser power. Nuclei were repeatedly photoactivated with 0.5-ms pulses with the total photoactivation time ranging from 0.5 to 120 ms. GFP fluorescence was imaged with a GFP filter cube. Average spot intensities and SD intervals (dark and pale lines, respectively; n=3 to 4 cells) are plotted as a function of the laser exposure time. (b) U2OS-PAGFP-H2A cell nucleus (arrow) after photoactivation (1 ms; 7.55 mW). Fluorescence intensity is visualized as a heat map. (c, d) Profile plots of PAGFP in fixed cells (N=4) (c) and in live cells (N=4) (d) after photoactivation. (e) Photoactivated GFP intensity in live cells as a function of GFP excitation time to show the bleaching rate (N=5).

To assess the capabilities of the DOE photoactivation system in cell biology assays, we generated a stable osteosarcoma (U2OS) cell line expressing PAGFP fused to histone H2A (PAGFP-H2A). Cells were cultured on glass-bottom 35 mm dishes. First, laser intensity and pulse duration were varied to optimize PAGFP photoactivation while minimizing phototoxic damage.^8^ As shown in Fig. 4(a), laser powers above 7.55 mW resulted in rapid photobleaching of PAGFP, as evidenced by the drop in PAGFP intensity after 20 and 30 ms of cumulative exposure to 9.94- and 11.70-mW peak laser powers, respectively. Laser powers were measured as indicated in Sec. 2.2. The cumulative exposure times were calculated by multiplying the pulse width of a single pulse (0.5 ms) by the number of identical pulses that were fed to the modulation port of the laser from a programmable function generator. Line profiles of the dot matrices indicated that short photoactivation times (<10  ms) were needed to minimize photoactivation outside of the intended matrix. This undesirable photoactivation is likely caused by 405-nm light scattering. We anticipate that short photoactivation times have the added advantages of minimizing phototoxic effects. These results and the photodamage constraints indicated that 1-ms pulses of 7.55-mW laser power were most suitable for chromatin tracking experiments. With these settings, spot arrays generated in fixed PAGFP-H2A cells had an FWHM of 560±70  nm [Figs. 4(b)–4(c)], which is 2.8 times the limit set by diffraction of PAGFP emission light (λ/2  NA=200  nm). Similar FWHM values (630±100  nm) were obtained with live cells [Fig. 4(d)].

**Fig. 5:**
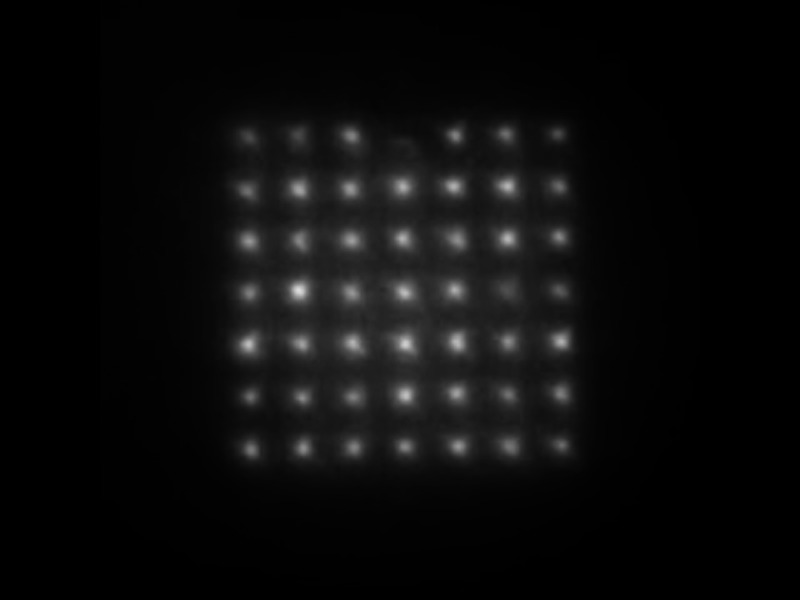
Time lapse imaging of a fixed U2OS cell expressing PAGFP-H2A (Video 1, MP4, 756 KB [URL: https://doi.org/10.1117/1.JBO.23.5.056007.1]).

**Fig. 6:**
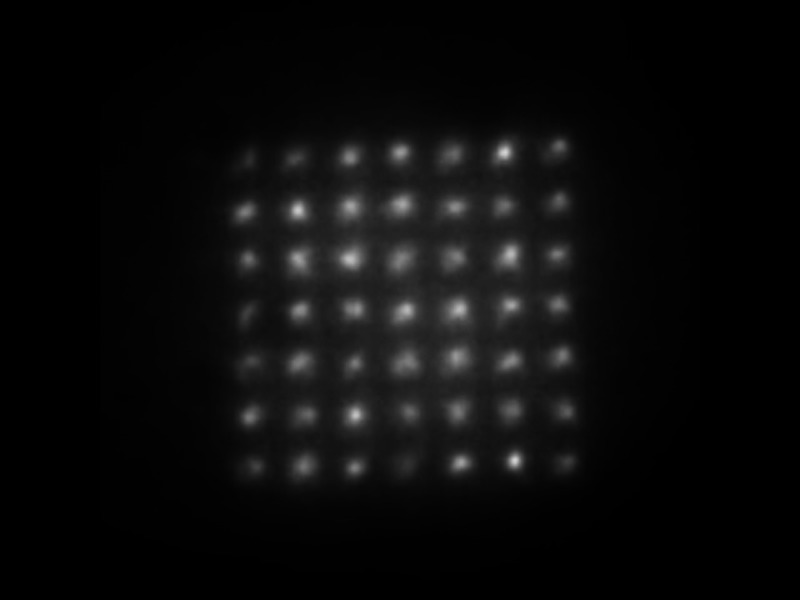
Time lapse imaging of a live U2OS-PAGFP-H2A cell (Video 2, MP4, 756 KB [URL: https://doi.org/10.1117/1.JBO.23.5.056007.2]).

To acquire time-lapse datasets for measurements of chromatin diffusion (D), the environmental chamber of the microscope was set to 37°C, and photoactivated PAGFP-H2A spots were imaged by epifluorescence microscopy in live cells for 1 min at a 3.16-fps frame rate. Imaging conditions were optimized to minimize the bleaching rate [Fig. 4(e)]. For comparison, cells were fixed with paraformaldehyde and imaged identically to live cells. Each dataset consisted of 200 frames. Representative time-lapse recordings of fixed and live cells (after registration, see below) are shown in Figs. 5 and 6, respectively.

### 3.2 Chromatin Tracking

**Fig. 7:**
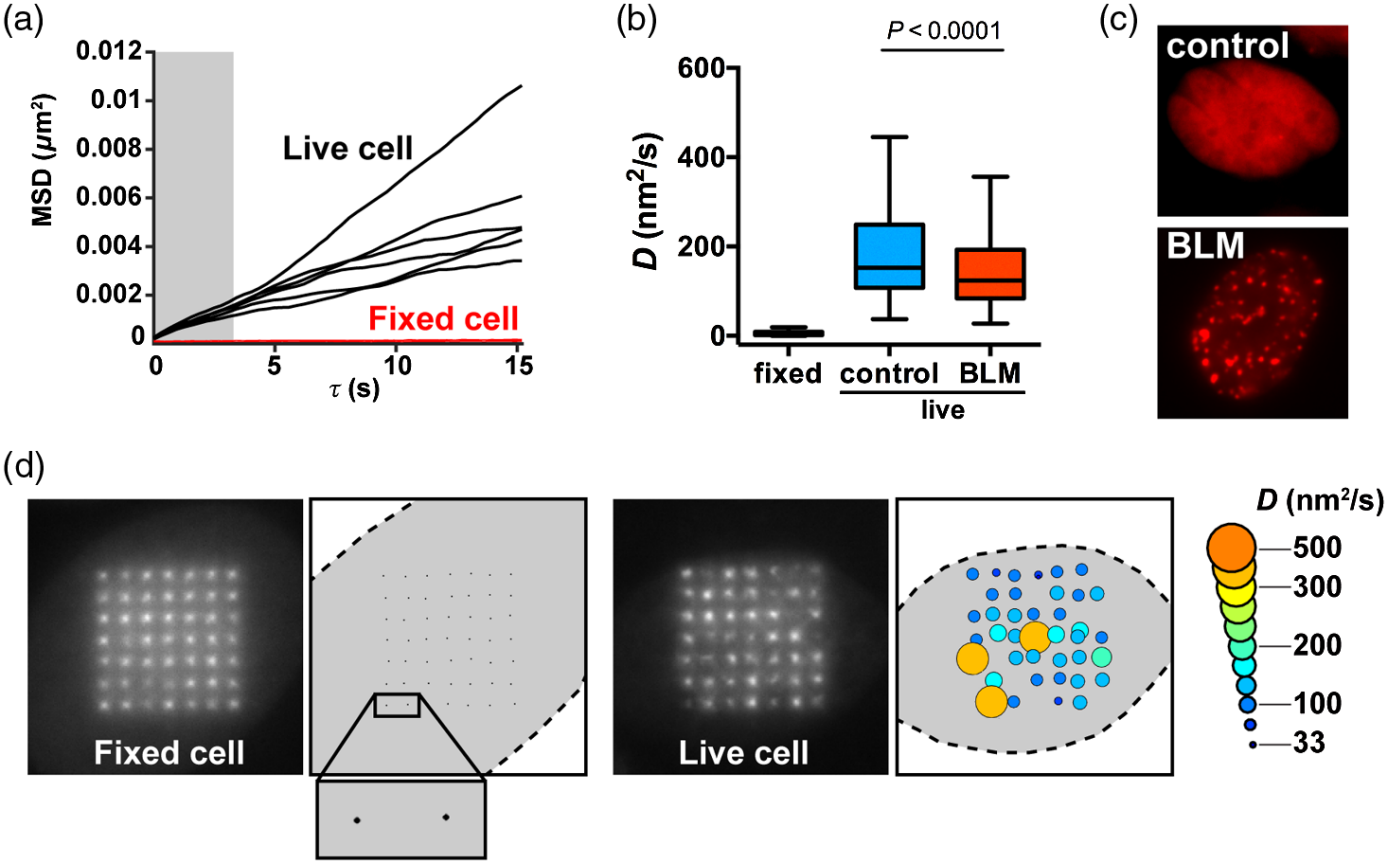
Mapping of chromatin motions in adherent cells. (a) Representative MSD curves used to evaluate D. Data are shown for six spots in one live cell (black lines) and six spots in one fixed cell (red lines). D was obtained from the slope of the MSD, using the first 12 values of τ (grayed region of the graph). (b) Box-and-whisker plot (Tukey method) of chromatin diffusion (D) measured in fixed cells (N=685 measurements from 19 cells) or in live cells. Live cells were either untreated (control; N=290 measurements from eight cells) or treated with the chemotherapeutic drug bleomycin (BLM, 20  mU/ml for 1 h; N=373 measurements from 10 cells). (c) Representative images of the DNA damage marker mCherry-53BP1ct in nuclei from untreated cells and from cells treated with BLM. (d) Maps of chromatin motions in fixed and live cells. The amplitude of D is visualized at specific nuclear regions with the size and colors of the circles. Small motions for fixed cells are visualized in the inset.

Cell migration as well as slight drifts of the microscope stage can confound measurements of chromatin motions. Hence, translations and rotations of the cell nucleus were first removed by sequential registration of the PAGFP-H2A images using the rigid body transformation of the StackReg plugin^19^ for ImageJ. We determined that this registration step does not influence relative spot positions. Next, using in-house MATLAB^®^ code, background fluorescence was subtracted as in Fig. 3. Importantly, this step removed photoactivated spot asymmetries, thereby improving Gaussian fits. Then, spots were tracked following the general approach of Crocker and Grier^20^ but with modifications. Since the spots were in a known pattern, each image was divided into 49 regions of interest, each containing only one spot. This made data analysis more robust. The center of each spot was then localized to subpixel precision in each frame by fitting the spot intensity to a 2-D Gaussian function.^21^ Finally, the diffusion coefficient D of each spot was evaluated from the slope of the first 12 points in the mean-squared displacement (MSD) curves (0.30 to 3.6 s). The 2-D MSD is defined as

$$\mathrm{MSD}(\tau )=<{\left[x(t+\tau )-x(t)\right]}^{2}+{\left[y(t+\tau )-y(t)\right]}^{2}{>}_{t}=4D\tau ,$$ (1)

where τ is the time gap between positions and t is the time. The symbol ${\left\langle \ldots \right\rangle }_{t}$ represents the average over all times collected. Assuming the time between frames is T (30 ms with our imaging setup), and given that 200 frames were collected, the first value of MSD can be computed for τ=T from 199 time gaps according to Eq. (1). The MSD for τ=2T is calculated similarly, but for 198 time gaps, etc. As expected, averaged D values were significantly higher (by a factor of 25), and more heterogeneous, in live compared to fixed cells [Figs. 7(a) and 7(b); F test of variance, P<0.0001]. Importantly, values of D measured with the DOE system in live cells closely matched previous measurements of chromatin diffusion in mammalian cells performed with different methods.^22^

We reported previously that DNA damage causes a transient decrease in chromatin diffusion in U2OS cells.^23^ This effect was clearly apparent when comparing D values from cells treated with the radiomimetic drug bleomycin (BLM) to control, untreated cells [Fig. 7(b)]. DNA damage induction by BLM was verified by expressing a DNA damage reporter (the C-terminal fragment of 53BP1 fused to mCherry; mCh-53BP1ct) [Fig. 7(c)]. This marker was used to select cells for analysis. Spatial distributions of D values were visualized in fixed and live cells using bubble maps [Fig. 7(d)]. The maps revealed unexpected levels of heterogeneity of chromatin diffusion at different locations in the cell nucleus. Follow-up studies will combine these maps with other spatial information of the cell nucleus (e.g., chromatin condensation, radial position, localization of nuclear bodies, etc.).

**Fig. 8:**
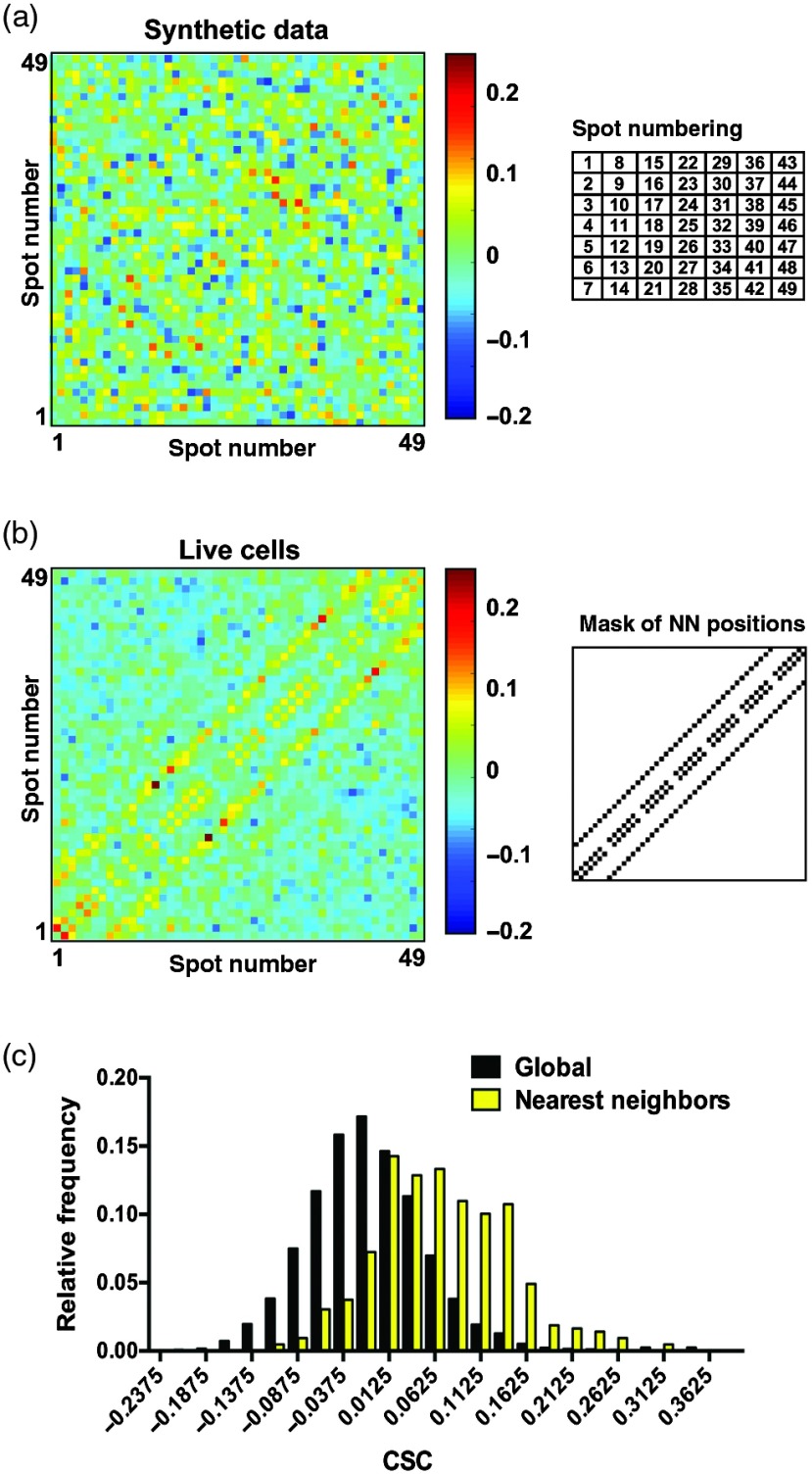
Correlation analysis of chromatin microdomain motions. (a) Heat map of the CSC for synthetic data. Each spot executes independent Brownian motion of amplitude similar to that in live cells. The simulated “pedestal” is a single Gaussian with radius and amplitude approximating the background seen in live cells (seven synthetic datasets). The spots are numbered from n=1 (upper left) to n=49 (lower right), as shown in the grid schematic. (b) Heat maps of the CSC in live cells (seven cells). A mask of the positions of NNs in correlation maps is shown on the right. NN within a column (n=1 to n=2; n=2 to n=3; etc.) generate the two lines close to the centerline. NN between columns (n=1 to n=8, n=2 to n=9, etc.) generate the two lines more distant from the centerline. Note the similarity of the regions of high CSC with NN positions. (c) Probability distributions of the CSC in live cells. Black bars: global distribution. Yellow bars: NN distribution.

Next, we determined if chromatin motions are coherent at the microscale level of our analyses. The cosine similarity coefficient (CSC) was used to assess whether the motions of two particles are correlated in direction. The CSC is defined as

$$\mathrm{CSC}=\frac{\overset{\to }{a}\cdot \overset{\to }{b}}{\left|\overset{\to }{a}||\overset{\to }{b}\right|},$$ (2)

where $\vec{a}$ and $\vec{b}$ are the displacement vectors of two different spots and $\left|\vec{a}\right|$ and $\left|\vec{b}\right|$ are the lengths of these vectors. Note that the CSC, like a correlation coefficient, has values in the range [−1,1]. Physically, the CSC captures how much the motion for one spot is along the direction of the motion of the second spot. The CSC is considered a useful metric for describing diffusive behavior in complex dynamical systems such as cells.^24^ The CSC was computed for all possible pairs between the 49 spots (N=1176 if all spots are trackable). First, seven synthetic datasets (cells) were generated, each consisting of 200 frames with 49 spots and including a background “pedestal”. Independent Brownian motion was assigned to each spot, moving the spot from frame-to-frame with a realistic diffusion coefficient. CSC values for the synthetic datasets were averaged and are plotted as heat maps in Fig. 8(a). The synthetic data showed little cosine similarity (or direction correlation) (CSC=0.0013±0.05; mean±SD, N=7506 spot pairs), as expected. CSC results were not affected upon altering values of radius (R=7 to 10) and height (H=2 to 10) applied in the “rolling ball” background subtraction step, indicating that this preprocessing step does not cause artifacts. Next, the CSC analysis was applied to live cell data [Fig. 8(b)]. A clear pattern of increased CSC is seen at the nearest neighbor (NN) positions. In Fig. 8(c), we show the distributions of CSC values for the live cell data [Fig. 8(b)] for all spots (global) and also for NN spots only. When considering all pairs of spots, no correlation was observed (CSC=−0.01±0.06, N=5825). However, NN spots showed clear correlations [CSC=0.07±0.08, N=428; Figs. 8(b) and 8(c)].

**Fig. 9:**
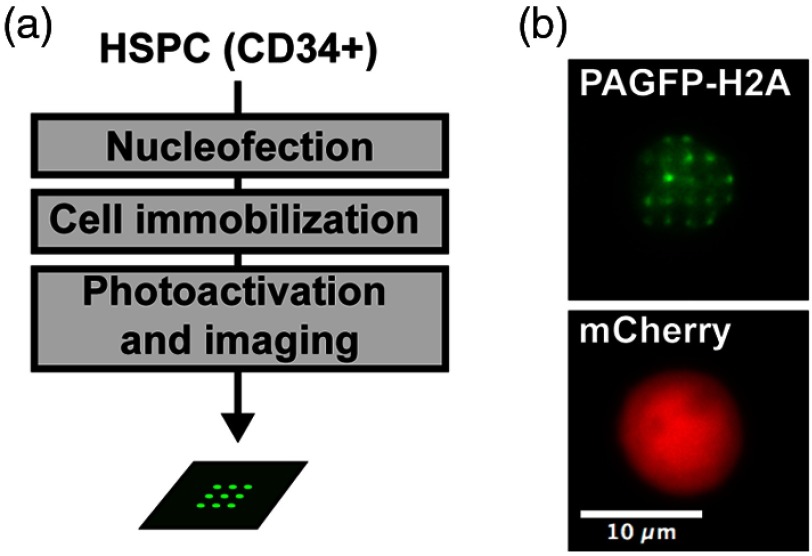
Chromatin motions in nonadherent hematopoietic stem/progenitor cells (HSPCs). (a) Flow chart of the procedure for sample preparation and data acquisition. (b) DOE photoactivation of PAGFP-H2A in HSPCs, giving ∼20 bright spots on a green background. Nucleofected cells were identified based on red fluorescent signals from mCherry, which was coexpressed with PAGFP-H2A.

Chromatin motions are necessary for the biogenesis of genomic translocations^25^ that drive hematologic malignancies. We therefore anticipate applications of the method presented herein for the study of chromatin dynamics in the context of the hematopoietic system, and developed a protocol for analysis of (nonadherent) $\mathrm{CD}34^{+}$ hematopoietic stem/progenitor cells (HSPCs) (Fig. 9). Briefly, fresh HSPC were maintained for 24 h in culture, then nucleofected with PAGFP-H2A and mCherry plasmids using an Amaxa protocol (Lonza). Thirty-hours later cells were immobilized on glass coverslips using a cell adhesive (CellTak, Corning) and embedded in a hydrogel topped with HSPC medium. Nucleofected cells are easily identified based on mCherry fluorescence, then PAGFP-H2A is photoactivated with the DOE system and time-lapse images recorded, as for the adherent U2OS cells.

## 4 Discussion

We have developed an approach based on structured illumination to quantify the movement of photoactivated microdomains of the cell with nanometer accuracy. One application of this method is illustrated by tracking chromatin motions. Based on spot sizes, the number of nucleosomes tracked collectively is estimated at $10^{5}$. Therefore, the method yields mesoscale readouts of global chromatin motions at specific subdomains of the cell nucleus, which are complementary to other tracking approaches where shorter stretches of chromatin are labeled. Beyond measurements of chromatin motions, we anticipate a broad application range of the method to measure diffusion, with the advantage of reducing phototoxic damage to cells compared to photobleaching-based techniques.^26^ Our method can simultaneously map distinct spatial regions, unlike FRAP and raster imaging correlation spectroscopy.^27^

Previously, a paired-particle tracking approach was implemented to determine chromatin D, using a confocal microscope for photoactivation and imaging of two PAGFP-H2A spots.^23^ With this pairwise method, or with a more recent extension of the confocal approach to nine spots (unpublished results), photoactivated spots could be tracked up to 1 min (at 3.3 fps). Then, photobleaching prevented tracking. The new DOE-based design has several advantages over these previous approaches. First, the initial intensities and positions of the photoactivated spots are well-defined. This reproducibility greatly eases analyses. Second, the method is simple. It involves a passive optical element that requires no adjustment. Third, it is well-modeled since each photoactivation beamlet has a known, quasi-identical intensity. Hence, intensity patterns detected in cells represent the expression level of the reporter in different cellular regions rather than illumination artifacts. The fourth advantage is speed. All spots are illuminated simultaneously. The fifth advantage is ease of integration. The DOE module can simply be mounted onto existing microscope platforms. Sixth, the current approach is flexible. Spot patterns can be changed by swapping one DOE for another. The seventh advantage is the high quality patterns produced by the DOE, which result in narrow spots (∼40% smaller than those achieved with a Zeiss CLSM710 confocal system). The final (eighth) advantage is the high spatial sampling capability of the DOE method, due to the added speed and spot resolution. We estimate a limit of sampling density of 1  μm based on calculated FWHM values, corresponding to 30 to 300 spots/nucleus depending on cell types, although this limit may eventually be limited by the extent of the chromatin motion. One limitation of the method is the possibility of vibrations from acoustical or mechanical sources causing pattern blur or distortion, in particular when the DOE module is mounted on a condenser mount. Vibration effects can be minimized to being negligible by quick laser pulsing and were found not to be an issue for short photoactivation times. A second challenge is that a small amount of scattered light produces background activation of the reporter that needs to be subtracted postimaging for optimal tracking results.

## 5 Conclusions

Maps of chromatin motions in live cells have been obtained by a method based on diffractive optics and photoactivatable chromatin reporters. The maps hint that correlated motions exist between adjacent focal volumes in chromatin microdomains at the time scale used for analysis. The imaging approach may lead to a better understanding of the mechanisms regulating chromatin dynamics in normal and pathological contexts. Application of this method to clinical samples will allow us to test the hypothesis that chromatin motions predict myeloid neoplasms caused by genomic translocations.
